# Coronary CT Angiography in the Emergency Department: State of the Art and Future Perspectives

**DOI:** 10.3390/jcdd12020048

**Published:** 2025-01-27

**Authors:** Antonio De Vita, Marcello Covino, Sara Pontecorvo, Giacomo Buonamassa, Angelo Giuseppe Marino, Riccardo Marano, Luigi Natale, Giovanna Liuzzo, Francesco Burzotta, Francesco Franceschi

**Affiliations:** 1 Faculty of Medicine and Surgery, Catholic University of the Sacred Heart, 00168 Rome, Italy; macovino@gmail.com (M.C.); sara.pontecorvo15@gmail.com (S.P.); giacomobuonamassa@gmail.com (G.B.); angelomarino1995@gmail.com (A.G.M.); riccardo.marano@policlinicogemelli.it (R.M.); luigi.natale@policlinicogemelli.it (L.N.); giovanna.liuzzo@policlinicogemelli.it (G.L.); francesco.burzotta@policlinicogemelli.it (F.B.); francesco.franceschi@policlinicogemelli.it (F.F.); 2 Department of Cardiovascular Sciences, Fondazione Policlinico Universitario A. Gemelli, IRCCS, 00168 Rome, Italy; 3 Department of Emergency Medicine, Fondazione Policlinico Universitario A. Gemelli, IRCCS, 00168 Rome, Italy; 4 Department of Diagnostic Imaging, Oncological Radiotherapy and Hematology, Fondazione Policlinico Universitario A. Gemelli, IRCCS, 00168 Rome, Italy

**Keywords:** coronary computed tomographic angiography, emergency department, acute chest pain, acute coronary syndrome, coronary artery disease

## Abstract

About 5% of annual access to emergency departments (EDs) and up to 25–30% of hospital admissions involve patients with symptoms suggestive of acute coronary syndrome (ACS). The process of evaluating and treating these patients is highly challenging for clinicians because failing to correctly identify an ACS can result in fatal or life-threatening consequences. However, about 50–60% of these patients who are admitted to the hospital because of chest pain are found to have no ACS. Coronary computed tomographic angiography (CCTA) has emerged as a proposed new frontline test for managing acute chest pain in the ED, particularly for patients with low-to-intermediate risk. This narrative review explores the potential of adopting an early CCTA-based approach in the ED, its significance in the era of high-sensitivity troponins, its application to high-risk patients and its prognostic value concerning atherosclerotic burden and high-risk plaque features. Additionally, we address clinical and technical issues related to CCTA use for triaging acute chest pain in the ED, as well as the role of functional testing. Finally, we aim to provide insight into future perspectives for the clinical application of CCTA in the ED.

## 1. Introduction

About 5% of annual access to the emergency department (ED) and up to 25–30% of hospital admissions involve patients with symptoms suggestive of acute coronary syndrome (ACS) [1]. The process of evaluating and treating these patients is highly challenging for clinicians because failing to correctly identify an ACS can result in fatal or life-threatening consequences [2]. However, about 50–60% of patients presenting with acute chest pain are found to have no ACS [3,4]. This over-triage carries considerable economic consequences, with related costs of around USD 14 billion for the United States healthcare system [5]. Furthermore, this overcrowding in the ED has been associated with worse clinical outcomes for acute chest pain patients, making rapid triage essential for both health and economic reasons [6,7]. From this perspective, coronary computed tomography angiography (CCTA) has been proposed to enhance decision-making in the ED for patients with no known previous coronary artery disease (CAD). Considering the high accuracy of CCTA in ruling out CAD, it allows a rapid evaluation of the degree of coronary atherosclerosis, drastically reducing the time-to-discharge of patients without significant CAD and ensuring more appropriate referral for invasive coronary angiography (ICA) and myocardial revascularization [8,9,10,11].

In this review, we explore the potential of an early CCTA-based strategy for patients with chest pain in the ED setting, its significance in the era of high-sensitivity troponins and its application for high-risk patients. Additionally, we discuss the prognostic value of the atherosclerotic burden, the high-risk plaque features and the role of functional testing in the acute setting.

## 2. Coronary CT Angiography-Based Approach in the Emergency Department

Extensive recent evidence and current guidelines support CCTA as a first-line imaging strategy for the rapid triage of low-to-intermediate risk patients presenting with acute chest pain to the ED (Table 1). The main advantages of an anatomical CCTA-based approach in the ED setting include its ability to (1) quickly rule out significant CAD in low-to-intermediate-risk patients thanks to its high sensitivity and negative predictive value; (2) shorten the time-to-diagnosis; (3) decrease the ED length-of-stay; (4) reduce the overall cost of care.

Firstly, data from the ROMICAT trial showed that, among patients presenting to the ED with acute chest pain and in whom the initial assessment was inconclusive, CCTA accurately excluded ACS in 71% of cases, demonstrating the absence of significant CAD (with a negative predictive value of 100%) [9]. Other studies suggest that a CCTA-based strategy for low-to-intermediate-risk patients presenting with suspected ACS allows for the safe and rapid discharge of about 50% of these patients, who would otherwise be admitted [8,9,10,11].

Secondly, both in the ROMICAT II trial and in the CT-STAT trial, the CCTA implementation in patients presenting with typical chest pain led to a 44% and 55% reduction in time-to-diagnosis, respectively, compared with rest/stress myocardial perfusion imaging (MPI) [12,13].

Thirdly, the ROMICAT II trial demonstrated that using CCTA significantly reduced ED length-of-stay by approximately 7 h and led to a higher rate of direct discharge from the ED, enhancing efficiency without compromising patient outcomes [13]. Similarly, in the CT-COMPARE trial, the implementation of CCTA was associated with a reduction in ED length-of-stay by approximately 6 h [14]. In all these studies, CCTA was used as a complement to clinical assessments, including troponins and risk scores. These parameters were crucial for stratifying patient risk and interpreting CCTA findings. Combining CCTA with traditional markers, rather than using CCTA alone, may enhance early triage and discharge decisions, providing a comprehensive patient evaluation and improving diagnostic accuracy. Furthermore, it is important to note that the study population primarily consisted of individuals with a low-to-intermediate risk profile and with a mean age under 60 years, which may influence the accuracy metrics. Across these studies, the sensitivity and specificity data are influenced by the prevalence of CAD, patient age and associated calcification levels. Older populations, with a higher burden of calcification, are more likely to experience higher false-positive rates. This context should be considered when interpreting the diagnostic accuracy metrics reported for CCTA in these trials and applying the result to older populations.

Lastly, in the CT-COMPARE trial, a CCTA-based strategy in patients presenting to the ED with acute chest pain led to a 20% reduction in hospital costs compared with the ECG exercise stress test [14]. This cost reduction encompassed all inpatient and outpatient costs associated with the index admission, including labor, diagnostics, pathology, pharmaceuticals, bed days and consumables, while excluding societal and opportunity costs. These findings were confirmed by the CT-STAT trial which demonstrated a 38% reduction in overall healthcare costs for patients assigned to the CCTA group compared to those in the MPI group [12]. Economic evaluations of CCTA often rely on key assumptions regarding the cost of the procedure, including reimbursement rates, operational efficiency and resource allocation within healthcare systems. These analyses also account for downstream cost savings from avoiding unnecessary hospital admissions, invasive procedures or additional testing. However, variations in local pricing, scanner availability and staffing requirements can significantly influence the economic outcomes.

Furthermore, CCTA offers several advantages compared to other diagnostic techniques available for managing patients with suspected ACS. In particular, it can detect the presence of other causes of chest pain (i.e., coronary artery abnormalities, aortic dissection and extra-cardiac causes), thus allowing clinicians to promptly initiate appropriate treatments [15,16].

### 2.1. Coronary CT Angiography in the Era of High-Sensitivity Troponins

Over the years, conventional cardiac troponin (cTn) tests have been largely replaced by high-sensitivity cardiac troponin (hs-cTn) assays in most centers. These hs-cTn assays have a higher negative predictive value (NPV), so normal levels can effectively exclude the presence of an ACS in ED patients presenting with acute chest pain [17,18]. However, the trade-off of this increased sensitivity is reduced specificity, meaning that hs-cTn assays can yield positive results not only in cases of myocardial infarction but also in various critical conditions associated with myocardial injury [19,20]. In these cases, the addition of CCTA beyond high-sensitivity troponin assays may be necessary to determine the underlying cause of chest pain and guide appropriate management.

In a study conducted in 500 patients, the use of CCTA was associated with less outpatient testing and lower direct medical costs compared to standard optimal care encompassing hs-cTn assays [21]. However, the BEACON study showed that CCTA did not improve the identification of patients, with significant CAD requiring coronary revascularization, nor did it reduce hospital stay or increase the rate of direct discharge from the ED [21].

Other studies provided promising evidence for the potential benefits of integrating CCTA into the ED setting, particularly for patients with intermediate levels of hs-cTn or without a significant “rise and fall” pattern [22,23].

The PRECISE-CTCA study showed that in patients presenting to the ED because of acute chest pain, in whom intermediate hs-cTn concentrations were found (between 5 ng/L and the sex-specific 99th percentile), there is a higher probability of CAD compared to those with low hs-cTn concentrations (<5 ng/L). In this specific subgroup of patients, CCTA may help identify those with occult CAD or with high-risk plaques, thereby improving clinical outcomes [24].

In a study by Ferencik et al., early advanced CCTA assessment (involving not only the degree of stenosis but also the plaque features) combined with hs-cTn evaluation may improve risk stratification and diagnostic accuracy in patients with suspected ACS and intermediate hs-cTn levels [25]. In these patients, the absence of coronary stenosis ≥ 50% and high-risk plaque ruled out ACS (ACS rate 0%), whereas patients with both stenosis ≥ 50% and high-risk plaque were at high risk for ACS (ACS rate 69.2%).

Finally, the COURSE trial is an ongoing study that aims to determine whether an early CCTA may be more efficient than standard care in patients with inconclusive high-sensitivity troponin results. Preliminary goals include reducing unnecessary hospital admissions and ICA [26].

### 2.2. Coronary CT Angiography for the Subgroup of High-Risk Patients

Although CCTA finds its greatest application in ED patients at low-to-intermediate risk, it might also be useful in high-risk patients presenting with acute chest pain.

Several studies have investigated the role of CCTA in patients presenting to the ED with a clinical picture of non-ST-segment elevation acute myocardial infarction (NSTEMI), demonstrating that CCTA may significantly reduce the need for ICA in a large number of cases [22,27]. It is especially advantageous for patients at greater risk of complications from invasive procedures, such as older individuals or those with a high risk of bleeding [27].

In the VERDICT trial, CCTA maintains high accuracy to rule out clinically significant CAD (defined as stenosis ≥ 50%) in patients with documented NSTEMI with a per-patient NPV of 91% [28]. Furthermore, it was observed that approximately one-third of patients with NSTEMI had no clinically significant CAD, indicating the potential role of coronary CTA to safely defer ICA in nearly one-third of these high-risk cases, with no increase in major adverse cardiovascular events (MACE) after a median follow-up of 4 years [28].

Other studies confirmed these results, showing that an early CCTA may reduce the need for ICA in NSTEMI patients by approximately 30 to 40%, compared with routine clinical care [29].

### 2.3. The Prognostic Value of Atherosclerosis Burden and High-Risk Plaques

**Figure 1:**
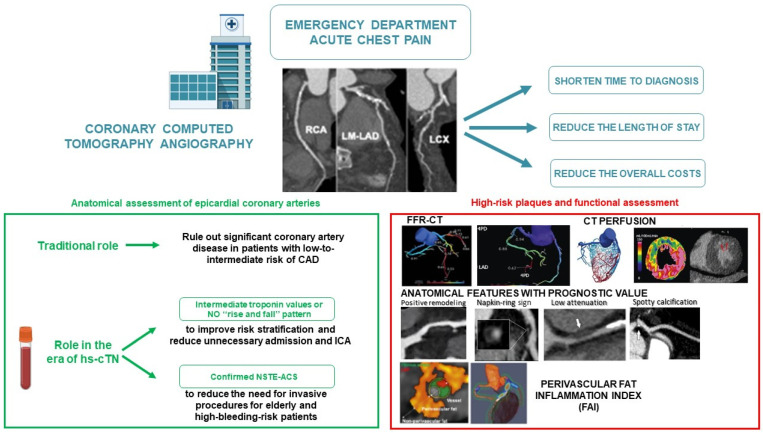
The role of CCTA in triaging patients with acute chest pain in the emergency department, integrating risk stratification, high-sensitivity troponin values, high-risk plaque characterization and coronary functional assessment.

Over the last few years, CCTA has gained additional prognostic value beyond merely assessing the severity of coronary stenosis. Indeed, CCTA performed in the ED setting also enables the evaluation of atherosclerotic burden and the presence of high-risk plaque features, both of which are associated with unfavorable outcomes (Figure 1).

The most compelling evidence linking atherosclerotic burden to unfavorable outcomes derives from the CONFIRM registry [30,31]. In another study by Bittencourt et al., the burden of coronary atherosclerosis, as documented by CCTA, has proven to be an independent prognostic factor in addition to the degree of stenosis: the segment involvement score (SIS) well correlates with survival (irrespective of stenosis severity), so an extensive plaque burden (SIS > 4) with non-obstructive plaque has the same risk for MACE as a lower plaque burden (SIS < 4) with obstructive plaque [32].

CCTA performed in the ED setting may be helpful to also identify high-risk plaque features, i.e., positive remodeling (>10% increase in vessel outer diameter), low attenuation plaque (plaque measuring < 30 HU), napkin-ring sign (plaque with a central area of low CT attenuation and peripheral ring of high CT attenuation) and spotty calcification (calcification ≤ 3 mm in any direction) [33].

Other features, such as increased plaque volume and fibro-fatty necrotic core, have been also associated with incident ACS: in the ICONIC trial both high-risk plaque features and quantitative assessment of fibrofatty necrotic core plaque volume were associated with incident ACS [34].

The perivascular fat inflammation index (FAI) has also been proposed as a potential marker for identifying high-risk plaque. The CRISP-CT study shows that higher pericoronary FAI values, indicating a higher inflammatory burden, are associated with a higher risk of adverse cardiac events [35]. Indeed, data from ROMICAT II showed that patients with high-risk plaque features on CCTA had an increased risk of ACS, independently of stenosis severity and clinical risk assessment [36].

Similar characteristics have been shown to indicate vulnerable plaque in histological studies (e.g., necrotic lipid-rich core, thin cap fibroatheroma, positive remodeling, spotty calcium) [37,38].

Although CCTA may help identify high-risk plaque with additional prognostic value, how plaque morphology influences decision-making in the ED setting remains unclear [39]. Further studies are needed to determine the optimal management strategies based on CCTA findings, particularly in patients presenting with acute chest pain.

### 2.4. Beyond the Anatomy: Functional Testing with Coronary CT Angiography

In recent years, CCTA has evolved, not only providing anatomical information about the degree of stenosis and high-risk plaque features but also offering functional insights about the physiology of lesion-specific ischemia and the hemodynamic impact of coronary stenosis using CT-derived fractional flow reserve (FFR-CT) and stress myocardial CT perfusion (CTP). This becomes particularly relevant in cases of intermediate coronary stenosis, where the presence or absence of myocardial ischemia may significantly influence the choice of therapeutic strategy [40,41].

FFR-CT has revolutionized the field of non-invasive cardiac imaging. Integrating data from CCTA with computational fluid dynamics algorithms, it estimates the functional significance of coronary artery lesions. This makes FFR-CT highly comparable to ICA in terms of both sensitivity and specificity [42]. In the ED setting, FFR-CT can provide valuable clinical insights in patients with suspected ACS. In a post hoc analysis of the ROMICAT II trial, in more than 50% of cases, stenoses assessed to be anatomically severe on CCTA were downgraded using the FFR-CT algorithm. Conversely, up to one-third of patients with mild stenosis (25–50%) were found to have hemodynamically significant flow limitation (FFR-CT < 0.80) [43].

Stress myocardial CTP is based on the distribution of iodinated contrast material during its first pass through the myocardium. It is possible to identify myocardial perfusion defects as hypo-attenuating areas since the amount of contrast material is reduced [44]. In patients presenting to the ED with acute chest pain, a combined stress CTP/CCTA strategy can result in fewer referrals for ICA compared to CCTA alone [45] and significantly reduced hospital costs and length-of-stay when compared to SPECT myocardial perfusion [46].

### 2.5. Patient Selection and Clinical Issues

Before performing CCTA in patients presenting to the ED with acute chest pain, it is crucial to stratify patients into different risk categories for ACS. There are different scores, such as the HEART pathway, EDACS score and ADAPT score, which can help to optimize the use of CCTA and ensure appropriate patient management [19,47].

The HEART pathway incorporates five key components: history, ECG, age, risk factors and troponin assessment. Patients are categorized into three risk levels: low risk (score < 3), intermediate risk (score 4–6) and high risk (score 7–10), based on the cumulative result of each item [48]. The EDACS score includes patient age, sex, cardiovascular risk factors and troponin serum levels [49]. The ADAPT score also incorporates the TIMI score, troponin measurement and ECG findings [50].

Numerous studies have suggested that CCTA in the ED setting is most effective in patients with chest pain and a low-to-intermediate risk for ACS [9,10,11,12], especially those with intermediate values of hs-cTn concentration (below the sex-specific 99th percentile), non-ischemic ECG changes or mildly abnormal functional testing [21,22,23,24]. In these groups, only a small percentage of patients have obstructive CAD, while most have normal or non-obstructive coronary arteries. Consequently, CCTA can be used effectively to rule out ACS and support the safe discharge of these patients.

However, CCTA may also be considered in patients with a high pre-test probability of CAD but without definite evidence of ACS by ECG and troponins, to confidently exclude CAD [25,26,27], especially in specific scenarios, such as patients at high bleeding risk or with vascular access issues. In patients with known CAD and prior PCI or prior CABG with normal ECG and normal hs-cTn, CCTA may be considered if certain criteria are met (e.g., stent in a proximal coronary segment and >3 mm in diameter) [47].

Based on the results of recent research, the 2023 ESC Guidelines for the management of ACS support the use of CCTA in ED patients with acute chest pain and non-elevated (or uncertain) hs-cTn levels, no ischemic ECG changes and no recurrence of symptoms (Table 1) [51].

### 2.6. Technical Issues

The integration of CCTA into the ED workflow requires careful consideration of several key aspects to ensure its safe and effective implementation. This includes a clear definition of indications, contraindications, patient preparation and optimization of the CT image acquisition [47,52].

The first step is defining whether the indication for performing CCTA is appropriate and screening patients for contraindications to contrast-enhanced CT (history of anaphylactic contrast reaction, inability to cooperate with breath-hold instructions, pregnancy, clinical instability and renal impairment) and for variables that may interfere with the image quality of CCTA (such as obesity, contraindication to betablockade, HR variability and arrhythmias) [53]. However, for patients with definite contraindications to contrast agents, a non-contrast CT scan for coronary calcium scoring may be considered [54,55].

Preparing patients for CCTA involves educating them on the importance of breath-holding during image acquisition to reduce respiratory motion artifacts. To obtain adequate images, a low and regular heart rate is necessary. The target heart rate reduction depends on the scanner’s temporal resolution, but generally, a target of 60–65 bpm or less is appropriate. Beta-blockers such as metoprolol (oral or IV) are commonly used to achieve HR reduction [56], with ivabradine as an alternative or adjunct [57,58]. Additionally, sublingual nitrates are used before CCTA to achieve coronary vasodilation and enhance coronary evaluation [59,60].

CCTA protocols should provide the highest diagnostic quality with the lowest radiation dose: all factors that influence the overall radiation exposure, such as the scanner type, tube voltage, tube current, scan range, scan acquisition time, gating (retrospective gating, prospective triggering), slice thickness and reconstruction method need to be optimized to minimize radiation exposure without compromising image quality [47].

### 2.7. The “Triple-Rule-Out” Protocol

Patients presenting to the ED with non-traumatic acute chest pain may have other potentially life-threatening conditions beyond ACS, such as aortic dissection and pulmonary embolism (PE). From this perspective, the introduction of “triple rule-out” (TRO) CT angiography represents a promising advancement in imaging technology, designed to assess at the same time coronary arteries, aorta and pulmonary arteries, with the potential to identify all three life-threatening etiologies of chest pain [61].

TRO CT angiography is particularly useful for ED patients with chest pain who simultaneously present risk factors for ACS (e.g., arterial hypertension, diabetes mellitus, smoking, dyslipidemia, obesity), PE (e.g., prolonged immobility, deep vein thrombosis, solid tumors) and aortic dissection (e.g., aortic aneurysm, aortic valve disease, Marfan syndrome) [62].

When TRO CT angiography is performed with appropriate attention to timing and technique, it yields image quality comparable to that of dedicated coronary CT angiography and pulmonary arterial imaging, thus potentially eliminating the need for further diagnostic testing in over 75% of patients [63]. However, in cases of strong suspicion of PE or aortic dissection, it would be preferable to conduct a dedicated CT angiography of the pulmonary arteries or aorta. This helps avoid potential diagnostic delays associated with TRO CT angiography.

TRO CT angiography is indeed conducted using an ECG-gated technique, resulting in a longer image acquisition time compared to a conventional CT scan. Therefore, information obtained from clinical history, physical examination, ECG and biomarkers (such as hs-cTn and D-dimer) may guide appropriate imaging technique selection.

Moreover, TRO CT angiography may involve greater radiation and contrast exposure compared to dedicated CT [64], with non-negligible clinical consequences.

In summary, whereas TRO CT angiography seems to be promising for the comprehensive evaluation of patients presenting to the ED because of chest pain, careful consideration of patient risk factors and clinical presentation is necessary to optimize its use and ensure timely and accurate diagnosis of life-threatening conditions.

### 2.8. Limitations of Routine CCTA Use in the Emergency Department

Although CCTA has emerged as a valuable tool for evaluating acute chest pain in the ED, offering high sensitivity for detecting obstructive CAD and a strong negative predictive value, its routine use poses several limitations that must be carefully considered (Table 2). One significant concern is the risk of overdiagnosis. CCTA frequently detects non-obstructive plaques or incidental findings of unclear clinical significance. These findings may lead to unnecessary diagnostic evaluations, increased healthcare costs and patient anxiety, with no clear benefit to patient outcomes.

Other limitations concern the use of iodinated contrast agents, as these can cause adverse reactions, including nephrotoxicity, particularly in patients with pre-existing renal impairment, and the exposure to ionizing radiation. Advances in low-dose protocols mitigate the latter risk but do not eliminate it entirely.

Furthermore, the diagnostic accuracy of CCTA can be considerably reduced in certain patient populations, such as those with extensive coronary calcifications, irregular heart rhythms or obesity, where image quality is often substantially compromised. CCTA also requires significant technical expertise and access to advanced imaging equipment, which may not be readily available in all healthcare settings. Accordingly, ensuring the availability of skilled CT interpreters, ideally accessible 24/7 to promptly review urgent CCTA scans, along with a scanner readily accessible on short notice and a dedicated CCTA nurse to administer beta-blockers and nitrates as needed, represents critical logistical considerations for effective implementation.

Lastly, integrating CCTA findings into clinical workflows requires careful consideration to avoid unnecessary interventions.

### 2.9. Future Perspectives

The future of CCTA in the ED setting extends beyond mere diagnosis, including risk stratification and prognostication. By identifying high-risk patients early, clinicians can implement appropriate interventions to prevent adverse cardiac events, ultimately improving patient outcomes. Given that most ACS events occur in patients with non-obstructive coronary lesions, focusing on identifying high-risk plaque features using CCTA may also provide additional risk assessment tools. Concerning this, an emerging role is played by the perivascular FAI indicating inflammatory burden and recently associated with a higher risk of adverse cardiac events [35,65].

Moreover, preliminary data suggest that the introduction of new CT algorithms, such as photon-counting CT, may provide images with very high resolution and low radiation exposure [66] and consequently reduce the rate of referrals for ICA [67].

The integration of artificial intelligence (AI) and machine learning algorithms may facilitate the analysis and interpretation of CT images, reducing the time-to-diagnosis and helping to start early appropriate management. Furthermore, high-resolution images pave the way for radiomics, encompassing the process of extracting numerous quantitative features from a given region of interest. These data may be used to identify new high-risk plaque features that can predict MACE [68].

The CT-derived left ventricular myocardial strain measurement represents another emerging area of research. It may help to find hypokinetic segments as an additional component in achieving an accurate diagnosis in suspected ACS patients [69,70].

Nonetheless, further studies are warranted to confidently deploy AI in clinical settings.

## 3. Conclusions

The main advantages of a CCTA-based strategy for patients presenting to the ED with acute chest pain include its ability to quickly rule out significant CAD in low-to-intermediate-risk patients, shorten the time-to-diagnosis, decrease the ED length-of-stay and reduce the overall cost of care. The early use of CCTA in the ED could also play a crucial role in specific high-risk patient subgroups, such as the elderly and those with bleeding issues. Furthermore, CCTA has gained additional prognostic value beyond merely assessing the severity of coronary stenosis. Indeed, CCTA performed in the ED setting also enables the evaluation of atherosclerotic burden and the presence of high-risk plaque features, both of which are associated with unfavorable outcomes. In recent years, CCTA has evolved, not only providing anatomical information but also offering functional insights about the hemodynamic effects of coronary stenosis, using FFR-CT and stress myocardial CTP. Finally, the introduction of new CT modalities combined with the integration of radiomics with AI and machine learning algorithms might enable CCTA to achieve even greater diagnostic and prognostic potential in the ED setting.

## Figures and Tables

**Table 1:** Recommendations from ESC and AHA/ACC guidelines on the use of CCTA for managing acute chest pain in the emergency department.

2023 ESC Guidelines for the Management of Acute Coronary Syndrome			2021 AHA/ACC/ASE/CHEST/SAEM/SCCT/SCMR Guideline for the Evaluation and Diagnosis of Chest Pain		
Recommendations	Class	Level	Recommendations	Class	Level
In patients with suspected ACS, non-elevated (or uncertain) hs-cTn levels, no ECG changes and no recurrence of pain, incorporating CCTA or a non-invasive stress imaging test as part of the initial workup should be considered.	IIa	A	For intermediate-risk patients with acute chest pain and no known CAD eligible for diagnostic testing after a negative or inconclusive evaluation for ACS, CCTA is useful for exclusion of atherosclerotic plaque and obstructive CAD.	I	A
Routine early CCTA in patients with suspected ACS is not recommended.	III	B	For intermediate-risk patients with acute chest pain with evidence of previous mildly abnormal stress test results (<1 year), CCTA is reasonable for diagnosing obstructive CAD.	IIa	C-LD
			For intermediate-risk patients with acute chest pain and no known CAD, as well as an inconclusive prior stress test, CCTA can be useful for excluding the presence of atherosclerotic plaque and obstructive CAD.	IIa	C-EO
			For intermediate-risk patients with acute chest pain and no known CAD, with a coronary artery stenosis of 40% to 90% in a proximal or middle coronary artery on CCTA, FFR-CT can be useful for the diagnosis of vessel-specific ischemia and to guide decision-making regarding the use of coronary revascularization.	IIa	B-NR
			For intermediate-risk patients with acute chest pain and known non-obstructive CAD, CCTA can be useful to determine progression of atherosclerotic plaque and obstructive CAD.	IIa	B-NR
			In patients with prior CABG surgery presenting with acute chest pain who do not have ACS, performing stress imaging is effective to evaluate for myocardial ischemia or CCTA for graft stenosis or occlusion.	I	C-LD

ESC: European Society of Cardiology; hs-cTn: high-sensitivity cardiac troponin; CCTA: coronary computed tomography angiography; ACS: acute coronary syndrome; AHA: American Heart Association; ACC: American College of Cardiology; ASE: American Society of Echocardiography; CHEST: American College of Chest Physicians; SAEM: Society for Academic Emergency Medicine; SCCT: Society of Cardiovascular Computed Tomography; SCMR: Society for Cardiovascular Magnetic Resonance; CAD: coronary artery disease; FFR-CT: computed tomography-derived fractional flow reserve; CABG: coronary artery bypass grafting; B-NR: non randomized; C-LD: limited data; C-EO: expert opinion.

**Table 2:** Contraindications and limitations of CCTA use in the emergency department.

CCTA for Triaging Acute Chest Pain in the Emergency Department	
**Contraindications**	
*Severe renal dysfunction*	Estimated eGFR below the threshold specified by local protocols, due to the risk of contrast-induced nephrotoxicity
*Known allergy to contrast media*	Severe hypersensitivity to iodinated contrast agents
*Hemodynamic instability*	Patients in shock or with acute respiratory distress, severe hypotension, unstable arrhythmia
*Lack of patient compliance*	Inability to cooperate with scan acquisition and/or breath-hold instructions
*Pregnancy*	Potential risks associated with fetal radiation exposure
*Uncontrolled tachyarrhythmia*	Heart rate too high for optimal imaging quality or inability to administer beta-blockers
*Severe obesity*	Patients exceeding scanner weight limits
**Limitations to routine use**	
*High coronary calcium score*	Extensive calcification, particularly in older patients, may reduce diagnostic accuracy
*Motion artifacts*	Difficulty maintaining breath-hold or body movement during the scan
*Limited availability*	Not all hospitals are equipped for emergency CCTA with trained personnel
*Contrast agent use*	Risk of nephrotoxicity or allergic reaction
*Previous PCI*	Artifacts from stents can impair the accurate assessment of coronary stenosis

CCTA: coronary computed tomography angiography; eGFR: estimated glomerular filtration rate; PCI: percutaneous coronary.

## Data Availability

No new data were created or analyzed in this study.
